# Remission after Six Months of Induction Immunosuppressive Treatment with Mycofenolate Mofetil and Prednisolone in Patients with Lupus Nephritis: A Descriptive Cross-sectional Study

**DOI:** 10.31729/jnma.6826

**Published:** 2021-08-31

**Authors:** Shailendra Shrestha, Rahul Yadav

**Affiliations:** 1 Nephrology Unit, Department of Internal Medicine, Nobel Medical College Teaching Hospital, Biratnagar, Nepal

**Keywords:** *immunosuppression*, *lupus nephritis*, *remission induction*

## Abstract

**Introduction:**

Treatment of lupus nephritis consists of six months of induction immunosuppression followed by years of maintenance immunosuppression. The aim of present study was to find the prevalence of remission after six months of induction immunosuppressive treatment with induction therapy in patients with lupus nephritis.

**Methods:**

A descriptive cross-sectional study was conducted in the nephrology unit of department of internal medicine of a tertiary care hospital from September 2018 to September 2020. The study was approved by institutional review committee of same institution (reference number: 184/2018). Convenience sampling method was used and Statistical Package for Social Sciences version 26 was used for statistical analysis. Point estimate at 90% Confidence Interval was calculated along with frequency and proportion for binary data.

**Results:**

Out of 24 patients, overall remission was seen in 21 patients (87.4%) (90% Confidence Interval= 76.26-98.54). Complete remission and partial remission were seen in 16 (66.6%) and 5 (20.8%) patients respectively resulting in an at the end of six months of induction immunosuppressive treatment. The most common class of lupus nephritis was class IV, 7 patients, followed by class IV+V, and class V, 6 patients in each respectively. The mean 24-hour urinary total protein, serum albumin and serum creatinine were 2492±1051 mg, 2.1±0.4 g/dl, and 0.9±0.1 mg/dl respectively. Adverse events were observed in 6 (25%) patients.

**Conclusions:**

Our study shows that good proportions of patients with lupus nephritis achieve clinical remission at the end of six months of induction immunosuppressive treatment with induction therapy, however, at the cost of some tolerable side effects.

## INTRODUCTION

Systemic lupus erythematosus (SLE) is a chronic systemic autoimmune disease and lupus nephritis (LN) is seen in about 40-50 % of the patients.^1,2^ The treatment of LN consists of six months of intense immunosuppression called induction treatment, followed by years of low dose maintenance immunosuppression. Either cyclophosphamide (CYC) or mycophenolate mofetil (MMF) along with prednisolone (PNL) are being used as an induction regimen in the treatment of class III, IV V, and mixed LN.^3-5^

There has been scarcity of data from Nepal regarding the clinical outcomes after six months of induction immunosuppressive treatment in patients with LN.^6,7^

Hence present study was conducted to find the prevalence of remission after six months of induction immunosuppressive treatment with mycophenolate mofetil and PNL in patients with lupus nephritis.

## METHODS

A descriptive cross-sectional study was conducted in the nephrology unit of the department of internal medicine of Nobel Medical College Teaching Hospital (NMCTH), Biratnagar, Nepal. All patients with LN from September 2018 to September 2020, who received six months of induction immunosuppression with MMF were included in the study. The study was approved by the Institutional Review Committee of the hospital (NMCTH IRC reference number 184/2018). Written informed consent was taken from all patients. The inclusion criterion was newly diagnosed patients of SLE with renal biopsy proven class III, IV, V and mixed LN. Lupus nephritis (LN) was classified according to the International Society of Nephrology/Renal Pathology Society (ISN/RPS) classification system.^8^ Patients were excluded if they had already received immunosuppressive therapy before enrolling in the study, if they disagreed to give the consent and when they failed to come monthly for at least six months. Convenience sampling method was used. The sample size was calculated using the formula:

n = Z^2^ × p × q / e^2^

  = (1.645)^2^ × (0.88) × (0.12) / (0.11)^2^

  = 23.6

  = 24

where,

n = sample size,
Z= 1.645 at 90% Confidence Interval,
p = prevalence, 88%,^6^
q = 1-p
e = margin of error, 11%

The calculated sample size was 24.

The baseline characteristics of the study population were digitally recorded at the time of enrollment in the study. After enrollment patients were started on induction immunosuppressive treatment for six months with MMF and PNL. Patients received MMF with a starting dose of 500 mg twice a day, which was increased after 2 weeks to 1000 mg twice a day for 6 months. Oral PNL was given with a starting dose of 0.5mg/kg for 2 months which was gradually tapered by 5 mg/every 2 weeks to reach 10mg/day. Injection methyl prednisolone (MP) 500 mg intravenous, once a day for three days was given at the start of induction regimen only if the serum creatinine was raised. Besides the immunosuppressive treatment, all patients also received angiotensin receptor blockers (ARBs) to titrate blood pressure below 125/75 mmHg, hydroxychloroquine 5mg/kg/day (maximum 400 mg/day), and a prophylactic antibiotic cotrimoxazole double strength (DS) ½ tablet once a day.

Clinical outcomes at the end of six months were assessed in terms of complete remission (CR), partial remission (PR) and no remission (NR) or deterioration, defined as per the Kidney Disease Improving Global Outcomes (KDIGO) guidelines.^9^ Complete remission (CR) was defined as return of serum creatinine to previous baseline, plus a decline in the urine protein creatinine ratio (uPCR) to <500 mg/g (<50 mg/mmol). Partial remission (PR) was defined as stabilization (±25%), or improvement of serum creatinine, but not to normal, plus a ≥50% decrease in uPCR.

Statistical analysis was done using Statistical Package for Social Sciences version 26. Point estimate at 90% Confidence Interval and descriptive statistics were done.

## RESULTS

Out of 24 patients, overall remission was seen in 21 patients (87.4%) (90% Confidence Interval= 76.26-98.54). Complete remission and partial remission were seen in 16 (66.6%) and 5 (20.8%) patients at the end of six months of induction immunosuppressive treatment. No response was seen in 3 (12.5%) patients (Table 1).

**Table 1:** Clinical remission after six months of induction immunosuppressive treatment with MMF^*^ and PNL^†^.

Remission	n (%)
Complete remission	16 (66.6)
Partial remission	5 (20.8)
Overall remission	21(87.4)
No response	3 (12.5)

* MMF: Mycofenolate mofetil;

† PNL:Prednisolone.

The mean age of the patients was 23.6±7.0 years, and 23 (95.9%) of them were females. The most common class of lupus nephritis was class IV, 6 (29.16%) patients, followed by class IV+V and class V, 6 (25%) patients in each class respectively (Table 2).

**Table 2:** Baseline characteristics of the patients (n=24).

Variables	n (%)
Gender	
Male	1 (4.1)
Female	23 (95.9)
Class of LN^*^	
Class III	3 (12.5)
Class III+V	1 (4.16)
Class IV	7 (29.16)
Class IV+V	6 (25)
Class V	6 (25)
Class V+III	1 (4.16)

* LN: lupus nephritis.

The mean 24-hour UTP, serum albumin and serum creatinine were 2492±1051 mg, 2.1 ±0.4 g/dl, and 0.9±0.1 mg/dl respectively. Baseline characteristics of the patients are shown in (Table 3).

**Table 3:** Clinicodemographic features.

Variables	Mean±S.D.
Age, years	23.6±7.0
IFTA^†^ (%)	11.0±2.0
24-hour UTP^‡^, mg	2492±1051
Serum albumin, g/dl	2.1±0.4
Serum creatinine, mg/dl	0.9±0.1
Hemoglobulin, g/dl	10.8±1.5

† IFTA: interstitial fibrosis tubular atrophy;

‡ UTP: urinary total protein.

Adverse events were observed in 6 (25%) patients during the six months of induction immunosuppression (Table 4).

**Table 4:** Adverse events during the six months of induction immunosuppressive treatment (n = 6).

Adverse events	n (%)
Herpes Zoster	2 (33.33)
Diarrhea	2 (33.33)
Tubercular plural effusion	1 (16.66)
Leukopenia	1 (16.66)

None of the patients died because of the adverse events.

## DISCUSSION

In the present study we analyzed the clinical remission rates and adverse events after six months of induction immunosuppression with mycophenolate mofetil and prednisolone in patients with lupus nephritis. We found that good proportions of patients achieve complete or partial remission at the cost of some adverse events.

The study done by Sedhai et al in Nepalese population had used NIH regimen 1.5 gm of mycophenolate mofetil along with prednisolone as an induction regimen.^7^ These are the only two published studies we could found from Nepal on extensive literature search, where the clinical outcomes were studied after six months of induction immunosuppressive treatment in patients with LN. Ours is the third study in the row.

Mycophenolate mofetil (MMF) as an alternative induction regimen in LN was introduced by Chen et al. in the year 2000; and the Aspreva Lupus Management Study (ALMS) established its role as a standard of care in the induction phase treatment of LN.^10,11^

In our study we used MMF in all our patients at a dose of 2 g/day (1 gm in two divided doses), which was less than the dose used in ALMS trial (3 g/day) but slightly more than the dose used in the Nepalese study (1.5 g/day).^7^-^11^

In the ALMS studies the response rate was 56.2% in the MMF group.^11^ In our study 66.6% of patients achieved complete remission and 20.8% of patients achieved partial remission. Overall remission rate was 87.4%. A higher rate of renal remission in our study as compared to western studies was also seen in previous studies done in Nepal.^7^ In another Nepalese study by Sedai et al,^7^ primary outcome (partial remission) and secondary outcome (complete response) were 28.6% and 66.7% in the mycophenolate mofetil group making an overall remission rate of 95.3 % in the MMF.^7^

Indeed, studies done in other South Asian populations have also shown better remission rates as compared to western studies.^12,13^ In the study done by Sahay et al. from India the overall remission rate was 72.9% in MMF group.^12^ In another Indian study by Rathi et al. total response rate was 74% in the low dose of MMF group; the complete remission rate was 54% in MMF group.^13^

In our study adverse events were observed in 25% of the patients, and they were herpes zoster and diarrhea in two patients each and tubercular plural effusion and leucopenia in one patient each. None of our patients died due to adverse events in our study.

The limitation of present study was that it was a single center study with a small number of participants. Furthermore, we only included patients who received MMF as an induction regimen and did not compared the outcomes with patients who received CYC. We recommend multicenter randomized control studies in Nepal comparing the two different induction immunosuppressive regimens.

## CONCLUSIONS

Our study demonstrates that good proportions of patients with lupus nephritis achieve clinical remission at the end of six months of induction immunosuppressive treatment with mycophenolate mofetil and prednisolone, however, at the cost of some tolerable adverse events.
